# Effect of Wearable Technology-Based Physical Activity Interventions on Breast Cancer Survivors’ Physiological, Cognitive, and Emotional Outcomes: A Systematic Review

**DOI:** 10.3390/jcm10092015

**Published:** 2021-05-08

**Authors:** Daphne S. Blount, Daniel J. McDonough, Zan Gao

**Affiliations:** 1 College of Biological Science, University of Minnesota–Twin Cities, 1445 Gortner Ave, Saint Paul, MN 55108, USA; Bloun025@umn.edu; 2 School of Kinesiology, University of Minnesota–Twin Cities, 208 Cooke Hall, 1900 University Ave SE, Minneapolis, MN 55455, USA; mcdo0785@umn.edu

**Keywords:** activity tracker, exercise, health promotion, randomized controlled trial, sedentary behavior

## Abstract

This systematic review synthesized all randomized controlled trials (RCTs) and controlled trials examining the effects of wearable health technology-based physical activity interventions on physiological, cognitive, and emotional outcomes in breast cancer survivors (BCS). We searched NCBI, Academic Search Premier, EMBASE, Web of Science, PubMed, and Medline from inception to March 2021. We included studies which: (1) were RCTs or controlled trials ≥8 weeks in duration; (2) were peer-reviewed and published in English; (3) sampled BCS in full remission and had not received treatment for at least six months; (4) utilized wearable health technology (e.g., Fitbit, Garmin xGC30); and (5) examined physiological, emotional, and/or cognitive outcomes. Sixty-six studies were identified and 14 were included in the review. Most of the observed effects were statistically significant and those which employed multi-component interventions generally yielded greater effects. Overall, the use of wearable health technology reduced sedentary behavior and increased moderate-to-vigorous intensity physical activity. Further, increased moderate-to-vigorous intensity physical activity was observed to be associated with increased perceived cognition and higher cognitive performance. Multiple studies also observed significant improvements in attitude, worry, and anxiety. Overall, findings suggested wearable health technology-based physical activity interventions to be effective for improving physical activity, attitude, and cognitive functions and for reducing sedentary behavior, anxiety, and worry in BCS.

## 1. Introduction

Breast cancer negatively affects millions of lives around the world as over 1.7 million new cases are diagnosed each year [1]. In the U.S. alone, there are currently over three million breast cancer survivors (BCS)—a number projected to reach four million in the near future [2]. Beyond the cancer itself, breast cancer is associated with other physiological, cognitive, and emotional changes that can negatively influence the health-related quality of life (HRQoL) of breast cancer patients and survivors [3]. Major risk factors associated with breast cancer in the physiological domain include sedentary lifestyles and associated chronic diseases such as obesity, hypertension, diabetes, and cardiovascular disease, to list a few [4]. Additionally, research indicates that approximately 78% of BCS also experience acute or chronic negative effects on cognitive function with many survivors reporting fogginess, loss of memory, attention deficit, and slowed cognitive function, among other perceived cognitive effects [5,6]. Lastly, breast cancer often has profound effects on survivors’ mental health and emotional wellbeing as depression, anxiety, fatigue, and stress are often observed at high levels in this population [6,7]. These three factors can individually or collectively influence a BCS’ HRQoL which can lead to a worsening of their overall health and create unnecessary risk for new or recurring diseases to develop.

Recent studies have identified BCS as a population at risk for the development of new health conditions due to a greater occurrence of the preceding risk factors [8]. Indeed, only 16% of BCS met the physical activity recommendations set by the U.S. Department of Health and Human Services of ≥150 min of moderate-intensity physical activity or ≥75 min of vigorous-intensity physical activity per week [9,10]. Additionally, this population also exhibits age-related risks, a higher prevalence of obesity, and many of the cognitive and emotional struggles previously outlined [5,6,8]. Thus, identifying innovative and effective physical activity promotion intervention strategies among BCS is an emerging public health challenge. Recent research has suggested this population is receptive to technology-based health interventions. In fact, a recent study by Delrieu et al. observed BCS to be willing and interested in the use of technology-based interventions to improve HRQoL outcomes, and furthermore, found these interventions to be effective for improving health behaviors such as physical activity participation [11,12]. Further, another study comparing various cancer survivor groups found that BCS, specifically, tended to have the greatest ability to improve physical activity behaviors during a technology-based intervention [8]. Not only are technology-centered interventions effective during experimental trials but a longitudinal follow-up study also observed improvements in HRQoL factors among BCS at 18 months post-intervention [13].

Previous studies have examined the effects of technology-centered intervention strategies on the three categories of wellbeing in BCS. Some examples include personal coaching, therapy, heart rate monitors, fitness trackers, calorie counting, social media platforms, emails, calls and text messages, smart scales, and smartphone applications. These interventions have been studied broadly, in the context of other diseases, and several of these studies have focused exclusively on BCS. However, concerns with several of these options have arisen regarding accessibility, safety, and efficacy [2,14,15]. Fitness trackers, or more broadly, health wearable tracking technologies (HWT; e.g., Fitbit, ActiGraph, Garmin xGC30), have emerged as a readily accessible, affordable, and effective option for physical activity and health promotion interventions among BCS.

The effects of HWT have recently been examined in several studies on the physiological [4,7,16,17,18,19,20,21,22], emotional [22,23,24], and/or cognitive [7,25] ailments that exist in BCS. Research employing HWT as a primary intervention component has suggested that this technology can be used as a successful medium by which to improve the preceding conditions among BCS [25]. One recent review noted that long-term weight loss was observed using HWT as an intervention component among overweight adults in some but all studies [26]. However, intervention adherence and bias prevented the review from providing a clear conclusion of this impact. Further, the application of HWT among BCS is a novel and emerging field of inquiry and there has been a recent surge in high-quality randomized controlled trials (RCTs) conducted examining the potential benefits of HWT interventions on this population’s physiological, cognitive, and emotional outcomes. Thus, there is a need for a review to systematically synthesize the current RCT- and controlled trial-based evidence on this topic to properly evaluate the overall effectiveness of HWT among BCS. In response, this systematic review of RCTs focused on measured health outcomes of BCS including physical activity, body weight, and composition, perceived cognitive clarity and function, and attitudes and preferences.

Though many trials have examined the use of HWT to promote physical activity and health among BCS, a systematic review has not yet assessed the overall efficacy and effectiveness of wearable health trackers as an intervention strategy to improve HRQoL among this population. Therefore, this systematic review aimed to discern whether HWT-based physical activity interventions are effective for enhancing the three quality of life domains among BCS—physiological, emotional, and cognitive. Further, this review aimed to establish if HWT is accessible and practical for use by BCS. Lastly, this systematic review aimed to establish a more current understanding of the findings in this field of inquiry, potential biases, areas for improvement, and future directions of investigation. Thus, we focused on BCS and the use of HWT as an intervention strategy to improve their HRQoL. By improving the understanding and applicability of using HWT in physical activity promotion interventions among BCS, clinicians and public health professionals may be better equipped to implement such health interventions to improve HRQoL and reduce the risk of further comorbidities among BCS.

## 2. Methods

The Preferred Reporting Items for Systematic Review and Meta-Analysis Protocols (PRISMA-P 2015) statement [27] guided the structure and reporting of this systematic review.

### 2.1. Study Eligibility Criteria

**Figure 1:**
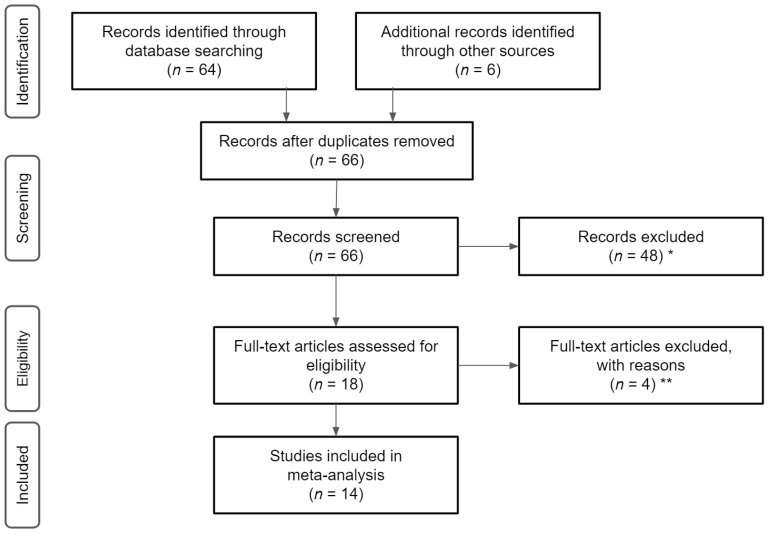
Flowchart of literature search and screening. Note, * reasons for exclusions included ineligible age, ineligible exposure, ineligible analysis, ** reasons for exclusion included ineligible outcomes and lack of means/standard deviations.

We applied the following criteria in the selection of studies for this review: (1) published in English prior to March 2021; (2) employed an RCT or controlled trial ≥8 weeks in duration; (3) used a sample of BCS who were six months post-treatment at the time of the study, must have previously received treatment for breast cancer but were not receiving treatment at the time of the study, who did not have any active form of cancer, and who had been at least two years post-diagnosis. We applied these strict criteria restrictions to ensure studies only included participants who were healthy and able to safely participate in the experiments and to avoid any confounds related to recent cancer and associated treatment, as well as treatment side effects; (4) used an HWT as an intervention component for physiological, emotional, and/or cognitive HRQoL factors. In detail, the studies must have included an HWT as the primary intervention component and this activity tracker must have been more advanced than a two-dimensional step counter (i.e., three-dimensional). Further, the articles must have included a tri-axial accelerometer that reported two or more measures of physical activity (e.g., moderate-to-vigorous physical activity, sedentary behavior), or a more advanced piece of technology that meets these minimum requirements; and (5) used quantitative measurements from the HWT to assess the health-related outcomes which we defined a priori [4,26,28,29,30,31,32]. Studies were excluded from this review if the participants did not meet the appropriate cancer- and cancer treatment-related criteria, the intervention strategy did not include the use of an HWT, the trial was not properly controlled, or if the article failed to meet any other inclusion criteria (See Figure 1).

### 2.2. Information Sources and Literature Search

The studies included in this literature review were all found electronically through one of the following databases or resources: NCBI, Academic Search Premier, EMBASE, Web of Science, PubMed, and Medline. The databases were searched for articles published prior to March 2021 to ensure we included the most novel and relevant HWTs. All databases were accessed through the study University’s library system. The advanced search feature, when available, was used to locate literature in the preceding databases. The following search terms were applied in logical orders to find potential literature for the review: breast cancer survivor, health wearable technology, health wearables, accelerometer, physical activity tracker, wearable activity tracker, physical activity sensing device, fitness tracker, sport tracker, smart watch, Fitbit, Garmin, and ActiGraph. Applicable literature was assessed for inclusion criteria. Acceptable literature was saved in PDF format and other relevant literature was also noted for use in the introduction and discussion. Other secondary literature searches (e.g., hand searching of reference lists of all articles, texts, and other review articles; clinical trials registry searches; unpublished literature through searches of conference proceedings; etc.) were conducted to ensure all relevant studies were considered.

### 2.3. Data Collection Process

The full texts of each article which met the preceding inclusion criteria were extracted separately by two investigators (DSB., DJM.; Table 1). Further, to characterize the included studies, we extracted information for each of these studies relevant to the following six topics: study goals and purpose, intervention methods, population details, methodology, results, and study main findings/conclusions. These data were sorted into a matrix table with similar studies grouped near one another. The studies were compared for information regarding each of the six preceding topics to assess similarities, differences, biases, and patterns, and this information was recorded in a Microsoft Excel spreadsheet. We employed descriptive and thematic analyses.

### 2.4. Data Items

The variables compared in this review were stratified into three overarching HRQoL-related domains: physiological outcomes, cognitive outcomes, and emotional outcomes. Namely, physiological outcomes included physical activity levels and body weight as well as oxygen exchange and body composition. Further, cognitive outcomes included perceived thought clarity, processing speed, and cognitive performance. Lastly, emotional outcomes included stress, anxiety, attitude and outlook, and cancer-related concerns. Variables that were measured in only one study were not included in data comparisons.

### 2.5. Risk of Bias within Individual Studies

Attributes of the Cochrane RoB2 Tool Guidelines and the Risk of Bias assessment used in Zeng et al. [26] were used to generate a nine-point assessment for evaluating the risk of bias in each of the individual studies. Similar categories from the Cochrane RoB2 tool were included and modified into a numerical format, as in Zeng et al. [26], to allow for quantitative comparison of risk of bias across all studies. Each study was assessed for each of these attributes and was ranked as either “+”, “−”, or “NA”. Specifically, “+” indicates that the attribute was explicitly presented in the literature, “−” indicates the attribute was not found in the literature, and “NA” indicates that the attribute was not applicable to the study. One point was awarded for each “+” per category while zero points were awarded for “−” or “NA”. These points were summed to generate a score for each publication based upon the ranking it received in each category. A low score (lower than the median score) suggested a heightened risk of bias, while a high score (higher than the median score) suggested the publication had appropriate protocol in place to reduce the risk of bias in the study. Articles with scores below the median score of 5 were reevaluated for inclusion by two study investigators (DSB., DJM.) and any discrepancies were adjudicated by a third investigator (ZG). Findings from articles with scores above the median score were considered more seriously in writing the conclusions of this review.

### 2.6. Summary Measures

Data recorded in the matrix and data with similar variables were directly compared. Quantitative data were used primarily for physiological outcomes and some cognitive measurements.

### 2.7. Synthesis of Results

Common themes in qualitative data were organized and the preceding bias-risk scores were considered when comparing the data. Due to the heterogeneity of variables collected and reported data types, a quantitative statistical comparison could not be conducted. Specifically, we did not employ a meta-analysis due to between-study heterogeneity in intervention types, intervention channels, study samples, and outcome measures, which made a meaningful analysis of pooled data impossible.

## 3. Results

### 3.1. Study Selection

Initially, 66 potential studies were identified using the above literature search procedure based upon the relevance to the subject. Of the 66 studies considered in the initial screening for this review, 14 were chosen based upon the strict inclusion criteria. The full texts of these articles, if available, were read to ensure they met the inclusion criteria.

### 3.2. Study Characteristics and Quality

Characteristics of individual studies are detailed in Table 1. Among the 14 included studies, all were conducted in a home setting. The U.S. was the primary location of the research with nine studies [4,7,16,17,21,22,34], while four studies were conducted in Australia [18,19,21,24], and one was conducted in Canada [20]. Nine studies in this review were published during or after 2018 [4,7,16,17,18,19,20,34], while only five studies were published between 2012 to 2017 [21,22,24], indicating the employment of HWT-based physical activity promotion interventions in BCS to be an emerging field of inquiry. There was large variability in sample size across studies which ranged from six to 358, with an average size of 89.2.

The sampling of most studies was convenience-based and largely dependent on the participant’s proximity to the location of the test site for intervention testing. Cancer treatment types varied widely and were unreported in most studies. Duration since treatment was at least six months prior to the beginning of the intervention in all studies and at least one year post-cancer diagnosis. However, recency of diagnosis and treatments varied between studies from six months to 10 years since treatment and one to 10 years since diagnosis. Cancer type was consistent across studies, namely breast cancer stages 1–3. Age of participants varied from 18 to 70 years, however, the predominant demographic were women 50–65 years, >90% of whom were Caucasian. However, one study focused specifically on African American women [22]. Several studies targeted recruitment at BCS who exhibited known risk factors or health characteristics, such as obesity [21], sedentary lifestyles [16,18,19,20,24,34], or being post-menopausal [18,19,24].

The wrist-worn wearable tracker of choice in the studies also showed variance. The Fitbit One [17,24], Polar A360/M400 [4,20,34], activPAL [18,19], and Garmin Vivofit [18,19,34] smartwatches were most popular as an intervention component. All but four studies [7,21,22,24] used the ActiGraph GTX3+ to take baseline measurements and other incremental measurements. Only four studies [16,18,34,35] utilized wrist-worn health wearables as an isolated intervention component while the other studies all included some other intervention component (i.e., a multi-component intervention).

### 3.3. Data Items

This literature review included studies that measured several different variables that impact HRQoL among BCS for the physiological, cognitive, and emotional domains. The physiological outcomes included physical activity, sit-to-stand transitions, body weight, and other biomarkers. The cognitive outcomes included perceived cognitive functioning, fogginess, speed of cognition, and memory. The emotional outcomes included participant’s feelings of anxiety, depression, fatigue, perceived emotional wellbeing, and their acceptance and preference for certain intervention styles or equipment.

### 3.4. Risk of Bias within Studies

The risk of bias within each individual study can be seen in Table 2. In detail, 10 studies were considered well controlled for bias based upon a score at or above five (i.e., the median score of all studies). The most common bias-reducing traits used in studies were randomization, used in 14 studies, the presence of a control group, used in 13 studies, and a baseline outcome measurement, used in 14 studies. Infrequently included was a six-month post-intervention follow-up, power analysis to determine sample size, and reporting of retention and adherence rates were also inconsistent.

### 3.5. Synthesis of Results

Among the studies which focused on a physiological trait as an outcome, methodology was similar across all studies in many ways. Most studies were a 12-week trial with baseline measurements taken by an ActiGraph GT3X+ as well as other biomarker tests done for seven days at a time at the week prior to the trial, six-week mark, and 12-week mark of the trial. Most control groups in the studies did not receive a fitness tracker, while the experimental group did receive the tracker as an intervention component during the trial. Many trials referred to two distinct methodology papers written for this field—Lynch et al. and Hartman et al. [3,5]. In studies that focused on cognitive functioning, these studies used the NIH Toolbox Cognitive Domain as the tool of assessment to measure cognitive outcomes. In the studies that addressed psychological and emotional impacts, the PROMIS measurement tool, also established by the NIH, was used most often. Many studies used patient databases and emailing or phone calls as the primary recruitment method for participants. In 10 studies, linear mixed models were used to assess the data and adjust for statistical error. ANOVA and bootstrapping were also used to assess variable relationships. Primary differences included the isolation or combination of interventions, the specific model of the wrist-worn activity tracker utilized, sample size, and differences in the definitions of physical activity.

### 3.6. Effectiveness of Health-Wearable Interventions

The effectiveness of the health-wearable trackers as an intervention component was assessed based upon statistical significance and trends of the results in the studies included in each category. Most of the positive relationships observed were statistically significant [7,16,17,18,19,20,21,22]. There were a handful of studies that reported insignificant improvements and it was suggested these relationships be further studied against more strict control [4,34]. Studies which utilized multi-component interventions generally yielded more significant results [18,19,20,21,22,33].

#### 3.6.1. Physiological Effects

Physiological health outcomes were the most heavily focused on HRQoL factors among the 14 included studies. Physical activity levels were objectively measured using triaxial accelerometers in 11 studies [4,7,16,17,18,19,20,21,22] which was the most popular variable measured, followed by sedentary behavior [16,18,19,20]. Additionally measured were participants’ body weight, body mass index, other blood-related biomarkers, physical fatigue, energy expenditure, and perceived physical health. Physical activity was hypothesized to have a positive effect on both cognitive function and emotional wellbeing. Overall, health wearable trackers tended to reduce sedentary behavior and increase moderate-to-vigorous intensity physical activity [4,16,17,18,19,20,21,33]. Improvements in other HRQoL factors, such as perceived wellbeing were also observed [20,21].

#### 3.6.2. Cognitive Effects

Efficacy in using health wearables to improve cognitive function was supported only when in association with another factor, most notably increases in physical activity and reductions in sitting time. Indeed, in studies which focused on increasing physical activity, cognitive functioning showed significant improvements, based upon the NIH Toolbox Cognition Domain measurement tools [7,34]. More specifically, increased moderate-to-vigorous intensity physical activity was observed to be associated with increased perceived cognition and higher cognitive performance scores among BCS [34]. Another study also suggested that increased MVPA could improve cognitive performance if mediated by a reduction in anxiety [7].

#### 3.6.3. Emotional Effects

Of the HRQoL factors, emotional wellbeing was the least researched and had the greatest variance in measured outcomes. One subtle psychological behavior studied was the frequency within which the participants would check their fitness tracking watches [17]. This study found that increased frequency of checking one’s activity data was associated with increased awareness and resulted in the participants developing positive attitudes about engaging in moderate-to-vigorous intensity physical activity and the use of the health-wearable technology [17]. However, checking moderate-to-vigorous intensity physical activity data on a phone or computer did not yield the same positive attitude results. One study assessed participants’ feelings towards the use of health-wearables as an intervention component and presented generally positive views on the technology and its efficacy for improving health, though this was a non-significant trend observed in sample sizes of 14 BCS [34]. The most common criticisms were how functional the wearable technology use is in participants’ busy lives, the ability of the user to understand the technology, and if seeing the data would have an overall negative effect on mood.

Aside from direct perceptions about the technology, a few studies focused on other measures of emotional wellbeing. In the aforementioned study that associated anxiety with cognition, there was evidence to believe that increased physical activity had the ability to directly reduce anxiety, which may result in improvements in cognitive performance [7]. While the data on the emotional outcomes were not observed statistically significant, these correlations were suggested by two other studies to have practical clinical significance included in the review [4,21].

## 4. Discussion

This literature review assessed the physiological, cognitive, and emotional health outcomes of using HWTs as the primary intervention component among BCS. The key outcomes that were of focus in most of the research were moderate-to-vigorous intensity physical activity, sedentary behavior, cognitive function, perceived cognitive ability, anxiety, and overall attitudes towards health-wearable technology. The key relationships that were suggested by the body of literature were as follows: (1) HWT has the potential to increase moderate-to-vigorous intensity physical activity, decreasing sedentary behavior, and increasing time spent standing among BCS; (2) increases in moderate-to-vigorous intensity physical activity may be associated with improvements in perceived and measured cognitive functioning while increases in bouts of sitting may be associated with a decline in cognitive functioning; (3) HWT may lead to improved attitudes about participants’ own health and wellbeing, reductions in anxiety, and improvements in HRQoL factors; and (4) health wearables are generally well accepted, easily learned and incorporated, and are perceived as being beneficial for improving one’s health. However, these technologies still have a few perceived barriers regarding accessibility, functionality, and undetermined long-term health benefits.

One of the goals of this review was to elucidate whether HWTs may be feasible for use in a clinical, preventative setting. Accessibility may be a barrier for some socioeconomically disadvantaged cohorts who may really benefit from this technology due to their often-increased risk factors and comorbidities [36]. For example, African American women have exacerbated rates of obesity (56%), cardiovascular disease (48%), and type 2 diabetes mellitus (13%)—risk factors which findings from this review suggest HWTs can significantly improve upon [36]. Several studies included in this review observed a positive correlation between HWTs and increased moderate-to-vigorous intensity physical activity. While this suggests improvements in physiological outcomes may have tangible clinical effects, further research should work to investigate this correlation in more rigorous and robust RCTs. Functionality of HWT has been validated by the high adherence rate of participants in the included studies to the use of the provided technologies. Furthermore, the preferences and attitudes towards using HWTs were largely positive. Very recent literature further validates that the BCS population is excited to use technology to improve health and views HWT as a functional method to facilitate this process [11,12]. Long-term health benefits should continue to be investigated, as many current studies only assessed an intervention period of 12 weeks. However, recent literature has suggested HWT to yield behavioral impacts in some populations for upwards of 18 months post-intervention [13,35]. Employment of long-term follow-up observations should be strongly considered for future studies in this field of inquiry [37]. Additionally, to further increase the effectiveness of such interventions, future research should consider integrating exercise training principles within their HWT-based physical activity interventions as suggested in recent BCS exercise research [38].

### Limitations and Recommendations

The practical implications of this review may have impacts on future research and public health preventative medicine practices to improve the health and HRQoL of BCS and many other groups of disease-afflicted people. This review provides information about the current practices and findings of HWT-based research with specific attention to BCS. However, research has already indicated that other demographics may benefit from similar interventions. Therefore, future research should work to clarify the information known about BCS, assess secondary outcomes which may improve health risk factors from a clinical perspective, and begin to explore new groups of chronically ill individuals that these technologies may benefit. It has been shown that fewer risk factors prior to breast cancer diagnosis allow for better recovery after treatment [9]. Given this knowledge, implementing HWT-based interventions as a preventative health strategy to promote physical activity and reduce risk factors may be beneficial for communities where the prevalence of heart disease, diabetes, and other risks are more common. Providing these communities with HWT to reduce their risk earlier in the lifespan may help to lower childhood chronic diseases, as well as prevent the development of other similar issues later in life. Additionally, using HWT in clinical settings to help patients who may be experiencing other cancers or diseases may aid in recovery as the body of BCS research has shown. 

There are several limiting factors to consider in the literature that was included in this review. Firstly, there was a lack of cognitively or emotionally focused studies. There were a plethora of studies focusing on physiological outcomes surrounding HWTs, however, there were effectively no studies that isolated the relationship of HWTs and cognitive function or psychological and emotional wellbeing. There were several relationships presented in this review that served as potential “next steps” to improve this field of HWT-based research. This direction of research should consider the reduction of sedentary behavior or increases in physical activity levels and the resultant improvement in cognitive function and reduced anxiety. With respect to the studies which evaluated BCS’ preferences and perceived barriers to HWT, the two studies that explored these perceptions did not have adequate sample sizes to draw reliable conclusions. However, these studies could be expanded upon in future research to determine the distinct advantages and barriers of HWT over other intervention types and whether or not these factors influence intervention adherence. Additionally, the multi-component aspect of this review was not designed to determine the benefits of providing BCS an HWT. Overall, the conclusions about the impact of the HWTs cannot be drawn without incorporating a more thorough analysis and integration of effects from other intervention components. Future literature reviews may consider statistically assessing the comparative effectiveness of HWT as isolated versus multi-component interventions to determine which intervention strategies/combinations are most effective for achieving the assessed outcomes. The emerging use of network meta-analysis in the field of physical activity and health promotion [39] would allow for such an assessment but future research in this field of inquiry should first aim to address the literature gaps we have identified in this review to allow for such comparisons. Further, greater consistency between studies would allow for better comparison of between-study results, including common outcome variable measures, intervention durations, randomized-controlled study designs, and greater sample sizes of BCS. Few papers at the time of our literature search met the criteria for inclusion, especially those evaluating emotional and cognitive effects. As future studies are conducted, a greater number of studies with adequate sample sizes and experimental designs may allow for more meaningful conclusions to be drawn. Notably, many of the studies included in our review had relatively small sample sizes. Indeed, only nine of the 15 studies [7,16,17,18,19,21,22,34] had sample sizes larger than 50 participants. Future studies would benefit by increasing their sample sizes to strengthen their findings and allow for more robust data analysis.

Diversity in population samples is another area in need of improvement. The majority of studies included in this review exhibited sample populations that were over 90% Caucasian and non-Hispanic. However, breast cancer effects women of different races at a greater proportion than what is currently being represented in most studies [25]. Only one study focused on a population of BCS that was not predominantly White and the data were based upon a combination of intervention strategies [22]. Thus, it is imperative to improve upon the diversity among participants because the demographic currently being represented is non-representative of the national or global population. Additionally, there are vastly different risk factors and accessibility barriers for women of different socioeconomic backgrounds that should be considered. One major advantage of HWT is its potential to serve as a preventative health intervention component in a very accessible and scalable way. Fitness trackers are comparatively affordable when considering the cost of successive cancer treatment or the treatment of other comorbidities that may arise. Evaluating the efficacy of HWTs among different demographics of BCS is necessary to determine if it is feasible for clinical applications.

Our risk of bias analysis observed that there are several factors of bias-reduction that can be improved upon across this field of research. Some of these factors include determining appropriate sample size, employing randomized-controlled study designs, and employing long-term follow-up assessments with participants after an intervention is complete. Additionally, isolating a single intervention to study (i.e., not employing multi-component interventions) may be useful in reducing potential confounds and may provide stronger evidence for the effects of HWTs directly on the measured outcomes. Improving these aspects of future studies would make the possible relationships derived from these data clearer. It is difficult to attribute a change in an outcome variable to one specific intervention strategy when there are several intervention strategies being applied at once. Furthermore, it is important to determine whether an intervention is sustainable outside of the circumstances of a study. Thus, follow-up data collection would be useful in determining if the participants were able to continue implementing the intervention successfully and yielding improvements in health-related outcomes after the trial has ended.

Lastly, there are limitations of the design and execution of this literature review that we must address. Firstly, we applied the inclusion criteria of only including studies of a minimum of 8 weeks in duration as we intended to strictly focus this review on interventions with similar intervention lengths that were observed common in the body of available research. While a recent repeat of the literature search did not reveal any studies of shorter duration that met our other inclusion criteria, this may have unnecessarily limited the breadth of studies included in this literature review. Further, it was difficult to compare the findings of all 14 studies included due to differences in sample size, reported data type, and variations in study design. Qualitative studies [23,24] identified trends in emotional outcomes regarding HWT use, however, it is not possible to objectively compare these findings without a more refined categorization process or quantification of these results. Additionally, Marinac et al. [35] was included in this review, though the activePAL device used in this study was worn on the thigh rather than the wrist, introducing an error in comparing the findings of this paper to those of strictly wrist worn HWTs.

The current body of research regarding HWT is rapidly evolving and it is imperative to continue research into their effectiveness in various clinical settings [40]. Research among BCS is showing promising potential for improving several HRQoL factors. These improvements in physiological, cognitive, and emotional health may be effective in also reducing risk factors for the development of other concurrent diseases—a common problem among BCS. HWT shows potential in improving physical activity, reducing sedentary behavior, reducing anxiety and worry, improving attitude, and improving cognitive functioning. However, improvements in study design including proper randomized-controlled designs and larger sample sizes are required in future studies to help to further elucidate the functionality of HWT. With these relationships emerging in very recent literature, continued inquiry in this field may yield clinical applications for BCS that may open doors to preventative and non-invasive health interventions for other diseases and demographics as well [40,41].

## Figures and Tables

**Table 1:** Characteristics of individual studies.

Title	Authors (Date)	Purpose and Intervention(s)	Sample and Setting	Design/Method	Findings
Effectiveness of Combined Smartwatch and Social Media Intervention on BCS Health Outcomes: A 10-Week Pilot Randomized Trial	Pope, Zeng, Zhang, Lee, Gao (2018) [4]	Goal: To determine effectiveness of combined smartwatch and social media-based intervention on BCS health outcomes. Intervention: Smartwatch combined with Facebook (FB) social media health education interventions.	30 middle aged BCS participants. Control group (*n* = 14) in FB group but did not have smartwatch, intervention group (*n* = 16) given Polar M400 smartwatch and in FB group. Mean age: 52.6 years old	10-week 2 arm randomized controlled trial. Outcomes measured at baseline and after 10-week trial. Physiological, psychosocial, and quality of life (QoL) factors measured by validated instruments. Physical activity (PA), energy expenditure (EE), and steps measured by ActiGraph GT3X+ and Polar M400. Primary end point: 10 weeks	Both groups had slightly increased daily LPA, ZMVPA, EE, and steps but there was no significant difference between groups. intervention group demonstrated improved sleep quality and reduced social life limitations. There were significant group differences for social support and barriers, control group improved in both areas, intervention group had decreased social support. Several BCS found the Polar M400 difficult to use.
Living Well after Breast Cancer: Changes in Objectively Measured Physical Activity in a Weight Loss Trial	Reeves, Winkler, McCarthy, et al. (2012) [33]	Goal: To determine if weight loss and PA are effective in improving BCS outcomes. Intervention: Accelerometer combined with telephone-delivered weight loss intervention.	90 overweight/obese women diagnosed with BC 9–15 months prior to study. Control group (*n* = 45) received usual care. Intervention group (*n* = 45) received behavior-focused weight loss interventions over the phone (goal >30 min MVPA per day). Mean age = 55.3 years old	Participants are from a convenience sample recruited from Queensland Cancer Registry. 6 month randomized controlled trial. GT3X+ ActiGraph measured PA at baseline and after 6-month intervention. Freedson cut-offs determined total MVPA per day. Daily lifestyle and walking/running MVPA identified using Crouter’s two-step regression. Linear mixed models used to examine intervention effects (only used for *n* = 983 valid days with 10+ h wear). Primary end point: 6 months	Both groups experienced significant increase in MVPA after trial, increase in MVPA was greater in intervention group than control group. Intervention significantly increased walking/running MVPA but not lifestyle MVPA.
A Randomized Controlled Trial of Wearable Technology-Based Intervention for Increasing Moderate to Vigorous Physical Activity and Reducing Sedentary Behavior in BCS: The ACTIVATE Trial	Lynch, Nguyen, Moore, Reeves, Rosenberg, Boyle, Vallance, Milton, Friedenreich, English. (2019) [19]	Goal: Use active smartwatch and other interventions to increase MVPA and reduce sedentary behavior in BCS. Intervention: Garmin Vivofit 2 activity tracker, behavioral feedback, goal setting, 5 telephone health coaching sessions.	80 inactive, postmenopausal BCS who had completed primary breast cancer treatment. Control group (*n* = 40) had no interventions, intervention group (*n* = 40) had described interventions. Age: ≥50 years old	12-week randomized controlled trial, GT3X+ ActiGraph and ActivPAL were used to measure physiological outcomes at baseline and after 12 weeks. Outcomes measured include PA and sitting and standing time. See Lynch et al. ACTIVATE methods paper for more information. Primary end point: 12 weeks	Significant change for both groups in MVPA with intervention group having greater MVPA increase. Significant change occurred in sitting time and prolonged bouts of sitting observed in both groups with intervention group having greater change from baseline.
Maintenance of Physical Activity and Sedentary behavior Change, and Physical Activity and Sedentary Behavior Change After an Abridged Intervention: Secondary Outcomes from the ACTIVATE Trial	Lynch, Nguyen, Moore, Reeves, Rosenberg, Boyle, Milton, Friedenreich, Vallance, English (2019) [18]	Goal: Determine if intervention from ACTIVATE trial had long lasting effects on MVPA and sedentary behavior after trial completion. Intervention: Garmin Vivofit 2, behavioral feedback, 5 telephone coaching sessions, goal setting	Participants from original trial, 80 inactive, postmenopausal BCS who had completed primary breast cancer treatment. Control group (*n* = 40) had no interventions, intervention group (*n* = 40) had described interventions. Age: ≥50 years old	MVPA and sedentary behavior were measured and compared from conclusion of 12-week trial to 12 weeks post-trial. MVPA and sedentary behavior were measured using ActiGraph and ActivPAL (worn for 7 days). Both groups received Garmin Vivofit 2 to use during post-intervention time. Linear mixed model with random effects were used to examine within-group changes in MVPA and sitting time. Primary end point: 12 weeks	12 weeks post-trial, women in intervention group had maintained increased levels of MVPA). At 12 weeks post-trial, sitting time had increased slightly but did not return to baseline. Control group when using smartwatch increased MVPA and reduced sitting time over 12-week post-intervention period.
Effects of The BEAT Cancer Physical Activity Behavior Change Intervention on Physical Activity, Aerobic Fitness, and Quality of Life in BCS: a Multicenter Randomized Controlled Trial	Rogers, Courneya, Anton, et al. (2015) [21]	Goal: Determine if BEAT Cancer intervention can change BCS PA, aerobic fitness, and QoL factors. Intervention: BEAT Cancer program vs. usual care. BEAT Cancer included supervised exercise, in-person counseling, group discussion, transition to home-based exercise.	222 post-primary treatment BCS. Control group (*n* = 112) did not receive intervention. Intervention group (*n* = 110) received BEAT Cancer program intervention. Age: 18–70 years old	3-month randomized controlled trial. Assessments measured at baseline, after 3-month intervention, and 3 months post-intervention. Baseline measurements made with use GT3X+ accelerometer and self-reported PA submaximal treadmill test, and QoL assessment as measurements. Adjusted linear mixed-model analysis used. Primary end point: 3 months	BEAT Cancer at 3 mo. showed significant increase in weekly minutes of MVPA (accelerometer measured) and self-reported exercise. Significant increase remained at 6 mo. only for self-reported PA. BEAT Cancer groups were significantly more likely to meet PA suggested standards at both the 3- and 6-month mark. BEAT Cancer significantly improved fitness at mo. 6 and QoL at 3 and 6 mo. BEAT Cancer intervention significantly improved PA, fitness, and QoL continuing up to 3 mo. post-intervention
Mediators of a Physical Activity Intervention on Cognition in Breast Cancer Survivors: Evidence from a Randomized Controlled Trial	Hartman, Weiner, Nelson, et al. (2019) [7]	Goal: Determine the mechanism through which PA influences BCS cognition. Intervention: ActiGraph GT3X+ accelerometer	87 sedentary BCS. Intervention group (*n* = 43) received the accelerometer and PA schedule. Control (*n* = 44) did not. Mean age: 57 years old	12-week randomized controlled trial. Outcomes measured include processing speed (NIH), self-reported cognition (PROMIS), physiological and psychological function (PROMIS), plasma biomarkers. Measurements collected at baseline and after 12-week intervention. Statistical methods include linear mixed-effect model and bootstrapping. See BEAT Cancer methods paper for more information. Primary end point: 12 weeks	Exercise arm group showed significant improvement in physical functioning and reduced anxiety. Anxiety significantly mediated intervention’s effect on cognitive abilities. Physical activity does not act as a mediator. No biomarkers had changes relevant to the study. Cognitive ability/processing speed did not have any mediated effect from PA.
Patterns of Fitbit Use and Activity Levels Throughout a Physical Activity Intervention: Exploratory Analysis from a Randomized Controlled Trial	Hartman, Nelson, Weiner, (2018) [17]	Goal: Explore the relationship between Fitbit activity trackers and success with PA interventions based on ActiGraph and determine if these interventions are sustainable. Intervention: Fitbit One worn daily throughout entire 12-week trial; ActiGraph used to create baseline and 12-week assessment	87 female BCS randomized into intervention and control arm groups. Control group (*n* = 44) did not receive a fitness tracker. Intervention group (*n* = 43) received Fitbit One, mobile app, website or combination thereof. Mean age: 57 years old	12-week randomized controlled trial. Data collected at baseline and at week 12. Fitbit One was worn daily to collect the metabolic equivalent of tasks (MET) on a per-minute basis all day. ActiGraph GT3X+ worn by both groups for 7 days at beginning and end to create baseline and 12-week assessments which measured the quantity of MVPA. Participants self-reported how frequently they checked their Fitbits using 8-point Likert scale. Mixed effects ANOVA used and linear mixed effects model. Primary end point: 12 weeks	Adherence to wearing Fitbit was high (~88%). Increased adherence to wearing Fitbit associated with increased MVPA (as measured by ActiGraph). 68% participants reported looking at tracker or website 1+ times per day. Changes in MVPA were significant with the wearable fitness trackers but not significant when checking app or website. More frequent checking of Fitbit associated to smaller changes in MVPA.
Breast Cancer Survivors Reduce Accelerometer-measured Sedentary Time in an Exercise Intervention	Weiner, Takemoto, Godbol, et al. (2019) [16]	Goal: Assess whether tech-based PA interventions can significantly increase MVPA. Intervention: ActiGraph accelerometer	87 BCS that were insufficiently active, were diagnosed less than 5 years prior to study enrollment, and had completed treatment. Control arm (*n* = 44) did not receive activity tracker while intervention arm (*n* = 43) did. Age: ≥21 years old	12-week randomized controlled trial. Outcomes measured were total sedentary behavior, LPA and MVPA. Outcomes were measured at baseline and after 12-week intervention using ActiGraph GT3X+ accelerometer. Linear mixed-effect models tested effects of intervention on sedentary, LPA and MVPA, change in sedentary vs. change in MVPA, and potential relationships between outcomes and sedentary behavior. Primary end point: 12 weeks	Exercise arm had significant reduction in sedentary behavior compared to control arm. Larger MVPA increase associated to larger sedentary decrease. Women further from surgery had significantly greater sedentary reductions. No significant changes in LPA.
Randomized Controlled Trial of Increasing Physical Activity on Objectively Measured and Self-reported Cognitive Functioning among Breast Cancer Survivors: The Memory and Motion Study	Hartman, Nelson, Meyers, Natarajan, Sears, Palmer, Weiner, Parker, Patterson (2018) [34]	Goal: Determine if intervention in PA would benefit cognition in BCS as compared to control group. Intervention: ActiGraph GT3X+ accelerometer	87 BCS, post-BC treatment, diagnosed at least 5 years prior to study, experiencing self-reported sedentary lifestyle or fogginess/depletion of cognitive function. Intervention group (*n* = 43) received accelerometer exercise intervention. Control (*n* = 44) did not. Age: ≥21 years old	12-week randomized controlled trial. Outcomes measured at baseline and at 12 weeks. ActiGraph measured PA. Objective cognitive performance was measured with the NIH Cognitive Toolbox. Self-reported cognition collected using Patient-Reported Outcomes Measurement Information scales. Linear mixed-effects regression model tested intervention and cognition change. Primary end point: 12 weeks	Processing speed showed significant improvement in exercise arm. Improvement in self-reported cognition between groups was not statistically significant but were large enough to show potential differences. Participants 2+ years post-surgery had significantly greater improvement in processing speed than participants less than 2 years post-treatment. Within the intervention group, those who had greater increased physical activity showed significant improvements in both objective and self-reported cognition.
Preventing Weight Gain in African American Breast Cancer Survivors Using Smart Scales and Activity Trackers: A Randomized Controlled Pilot Study	Valle, Deal, Tate (2016) [22]	Goal: Determine feasibility of using smart scales and activity trackers to prevent weight gain in African American BCS. Intervention: Withings WS-30 smart scale for daily weighing that sent data to app/website, emailed lessons, feedback on objective weight. For mixed group only, Withings pulse activity tracker.	35 AA BCS, diagnosed with stage 1–3 BC within the last 10 years, who have completed treatment, have no active cancer, and have a BMI of 20–45 kg/m^3^. There were two intervention groups and one control group. Group one (*n* = 11) received the activity tracker and other interventions. Group two (*n* = 13) did not receive the activity tracker, only the other interventions. The control group (*n* = 11) did not receive either. Age: ≥18 years old	6 month randomized controlled trial. Online and in-person assessments were taken at baseline, 3 month, and 6-month mark. Throughout the trial, the activity tracker and scale objectively measured PA and weight. Primary end point: 6 months	Average weight loss at 6 months was −0.9% in intervention + tracker group. Average weight loss at 6 mo. was −0.2% in intervention only group. Control group gained on average +0.2%. Adherence rate to trial was very high. Daily self-weighing was perceived as positive by both intervention groups and 100% of participants recommend program to other BCS.
A Qualitative Evaluation of Breast Cancer Survivors’ Acceptance of and Preferences for Consumer Wearable Technology Activity Tracker	Nguyen, Hadgraft, Moore, Reeves, Rosenberg, Lynch, Reeves, Lynch (2017) [24]	Goal: Assess the usability of wearable activity trackers among postmenopausal BCS. Intervention: random selection of six trackers (Fitbit One, Jawbone Up 24, Garmin Vivofit 2, Garmin Vivosmart, Garmin Vivoactive, Polar A300).	14 postmenopausal BCS women, stage 1–3 BC, post treatment completion at least 6 months prior. Age: ≥50 years old	Series of multiple 2-week randomized controlled trials. Women tested two or three randomly assigned trackers. The device was worn for 2 weeks with a 1-week break between wearing the next device. Each tracker had to count steps and notify the participants via a notification if they were sedentary for an extended period. Focus groups were held to interview women on perceptions and experience with each tracker. Thoughts were transcribed and grouped into themes and later analyzed statistically. Primary end point: 2 weeks	5 primary themes -Trackers increased self-awareness and motivation -BCS confidence and comfort with wearable tech -Preferred and disliked features of WAT -Concerns related to disease -Peer support and doctor monitoring presented as activity tracker applications
Activity Tracker to Prescribe Various Exercise Intensities in Breast Cancer Survivors	McNeil, Brenner, Stone, et al. (2019) [20]	Goal: Determine if activity tracker can be used to effectively prescribe various PA intensities and increase PA, reduce sedentary time, improve health outcomes. Intervention: Either low or high intensity PA intervention with a PA tracker (Polar A360).	45 BCS (survivors of stage 1–3 breast cancer) who are no longer in treatment (excluding hormone therapy), who currently take fewer than 10,000 steps per day. Intervention group one (*n* = 15) received low PA intervention with tracker (300 min/wk at 40–59% HR reserve). Intervention group two (*n* = 15) received high-intensity PA with tracker (150 min/wk at 60–80% HR reserve). Control group (*n* = 15) received no intervention. Age: ≥18 years old	12-week randomized controlled trial. Measurements recorded at baseline, 12 weeks, and 24 weeks taken with ActiGraph GT3X+ trackers. Outcomes measured were sedentary time, health-related fitness, and patient-reported outcomes. Intervention groups received Polar A360 trackers. Linear mixed model used to adjust data Primary end point: 12 weeks	In high intensity group, MVPA significantly increased. In low intensity group, sedentary time significantly decreased. VO_2_ Max was also significantly increased at 12-week mark for both experiment groups, with the high intensity group seeing a greater increase. Changes in PA and VO_2_ Max were sustained at 24-week follow up but were no longer significant.

**Table 2:** Assessment of risk of bias of individual studies.

Article	1	2	3	4	5	6	7	8	9	Total
Hartman et al. [34]	+	+	−	+	+	+	NA	+	−	6 *
Hartman et al. [17]	+	+	+	+	+	+	+	−	−	7 *
Hartman et al. [7]	+	+	−	+	+	+	−	+	−	6 *
Lynch et al. [19]	+	+	−	+	+	+	+	+	−	7 *
Lynch et al. [18]	+	+	−	+	+	+	+	−	−	6 *
McNeil et al. [20]	+	+	−	+	+	NA	NA	NA	+	5 *
Nguyen et al. [24]	+	−	+	NA	NA	−	+	−	−	3
Pope et al. [4]	+	+	−	+	+	−	NA	−	−	4
Reeves et al. [33]	+	+	−	+	+	NA	NA	−	−	4
Rogers et al. [21]	+	+	−	+	+	+	+	+	−	7 *
Valle et al. [22]	+	+	−	+	+	+	+	−	−	6 *
Weiner et al. [16]	+	+	+	+	+	+	NA	−	−	6 *

Note. Item numbers indicate the following quality: 1 = randomization of subjects, 2 = presence of a control in study, 3 = health wearable tracker technology was used as isolated intervention method, 4 = outcome variables were measured before and after intervention, 5 = baseline measurement was taken of key outcome variables, 6 = study retention was described and at least 70%, 7 = intervention adherence was described and at least 70%, 8 = power analysis was conducted to determine appropriate sample size, 9 = participants were followed up with for a minimum of 6 months post-intervention. Scores indicated: “+” = presence of attribute, “−” = absence of attribute, “NA” = not applicable to study. A * denotes a paper at or above the median score of 5 for bias-prevention methods.

## References

[1] Blaes A., Beckwith H., Florea N., Hebbel R., Solovey A., Potter D., Yee D., Vogel R., Luepker R., Duprez D. (2017). Vascular function in breast cancer survivors on aromatase inhibitors: A pilot study. Breast Cancer Res. Treat..

[2] Phillips S.M., Courneya K.S., Welch W.A., Gavin K.L., Cottrell A., Nielsen A., Solk P., Blanch-Hartigan D., Cella D., Ackermann R.T. (2019). Breast cancer survivors’ preferences for mHealth physical activity interventions: Findings from a mixed methods study. J. Cancer Surviv..

[3] Lynch B.M., Nguyen N.H., Reeves M.M., Moore M.M., Rosenberg D.E., Wheeler M.J., Boyle T., Vallance J.K., Friedenreich C.M., English D.R. (2018). Study design and methods for the ACTIVity And TEchnology (ACTIVATE) trial. Contemp. Clin. Trials.

[4] Pope Z.C., Zeng N., Zhang R., Lee H.Y., Gao Z. (2018). Effectiveness of Combined Smartwatch and Social Media Intervention on Breast Cancer Survivor Health Outcomes: A 10-Week Pilot Randomized Trial. J. Clin. Med..

[5] Hartman S.J., Natarajan L., Palmer B.W., Parker B., Patterson R.E., Sears D.D. (2015). Impact of increasing physical activity on cognitive functioning in breast cancer survivors: Rationale and study design of memory & motion. Contemp. Clin. Trials.

[6] Van Der Gucht K., Melis M., Ahmadoun S., Gebruers A., Smeets A., Vandenbulcke M., Wildiers H., Neven P., Kuppens P., Raes F. (2020). A mindfulness-based intervention for breast cancer patients with cognitive impairment after chemotherapy: Study protocol of a three-group randomized controlled trial. Trials.

[7] Hartman S.J., Weiner L.S., Nelson S.H., Hess L., Ehlers D., Dregan A., Natarajan L., Patterson R.E., Palmer B.W., Parker B.A. (2019). Mediators of a physical activity intervention on cognition in breast cancer survivors: Evidence from a randomized controlled trial. JMIR Cancer.

[8] Sweegers M.G., Boyle T., Vallance J.K., Chinapaw M.J., Brug J., Aaronson N.K., D’Silva A., Kampshoff C.S., Lynch B.M., Nollet F. (2019). Which cancer survivors are at risk for a physically inactive and sedentary lifestyle? Results from pooled accelerometer data of 1447 cancer survivors. Int. J. Behav. Nutr. Phys. Act..

[9] Boyle T., Vallance J.K., Ransom E.K., Lynch B.M. (2016). How sedentary and physically active are breast cancer survivors, and which population subgroups have higher or lower levels of these behaviors?. Support. Care Cancer.

[10] 2018 Physical Activity Guidelines Advisory Committee (2018). 2018 Physical Activity Guidelines Advisory Committee Scientific Report.

[11] Delrieu L., Vallance J.K., Morelle M., Fervers B., Pialoux V., Friedenreich C., Dufresne A., Bachelot T., Heudel P., Trédan O. (2020). Physical activity preferences before and after participation in a 6-month physical activity intervention among women with metastatic breast cancer. Eur. J. Cancer Care.

[12] Nelson S., Pialoux V., Pérol O., Hartman S., Delrieu L., Morelle M., Martin A., Friedenreich C., Febvey-Combes O., Pérol D. (2020). Feasibility and health benefits of an individualized physical activity intervention in women with metastatic breast cancer: Intervention study. JMIR mHealth uHealth.

[13] Fazzino T.L., Fabian C., Befort C.A. (2017). Change in physical activity during a weight management intervention for breast cancer survivors: Association with weight outcomes. Obesity.

[14] Walker R.K., Hickey A.M., Freedson P.S. (2016). Advantages and limitations of wearable activity trackers: Considerations for patients and clinicians. Clin. J. Oncol. Nurs..

[15] Kenfield S.A., Van Blarigan E.L., Ameli N., Lavaki E., Cedars B., Paciorek A.T., Monroy C., Tantum L.K., Newton R.U., Signorell C. (2019). Feasibility, acceptability, and behavioral outcomes from a technology-enhanced behavioral change intervention (Prostate 8): A pilot randomized controlled trial in men with prostate cancer. Eur. Urol..

[16] Weiner L.S., Takemoto M., Godbole S., Nelson S.H., Natarajan L., Sears D.D., Hartman S.J. (2019). Breast cancer survivors reduce accelerometer-measured sedentary time in an exercise intervention. J. Cancer Surviv..

[17] Hartman S.J., Nelson S.H., Weiner L.S. (2018). Patterns of Fitbit use and activity levels throughout a physical activity intervention: Exploratory analysis from a randomized controlled trial. JMIR mHealth uHealth.

[18] Lynch B.M., Nguyen N.H., Moore M.M., Reeves M.M., Rosenberg D.E., Boyle T., Milton S., Friedenreich C.M., Vallance J.K., English D.R. (2019). Maintenance of physical activity and sedentary behavior change, and physical activity and sedentary behavior change after an abridged intervention: Secondary outcomes from the ACTIVATE Trial. Cancer.

[19] Lynch B.M., Nguyen N.H., Moore M.M., Reeves M.M., Rosenberg D.E., Boyle T., Vallance J.K., Milton S., Friedenreich C.M., English D.R. (2019). A randomized controlled trial of a wearable technology-based intervention for increasing moderate to vigorous physical activity and reducing sedentary behavior in breast cancer survivors: The ACTIVATE Trial. Cancer.

[20] Mcneil J., Brenner D.R., Stone C.R., O’Reilly R., Ruan Y., Vallance J.K., Courneya K.S., Thorpe K.E., Klein D.J., Friedenreich C.M. (2019). Activity tracker to prescribe various exercise intensities in breast cancer survivors. Med. Sci. Sports Exerc..

[21] Rogers L.Q., Courneya K.S., Anton P.M., Hopkins-Price P., Verhulst S., Vicari S.K., Robbs R.S., Mocharnuk R., McAuley E. (2015). Effects of the BEAT Cancer physical activity behavior change intervention on physical activity, aerobic fitness, and quality of life in breast cancer survivors: A multicenter randomized controlled trial. Breast Cancer Res. Treat..

[22] Valle C.G., Deal A.M., Tate D.F. (2017). Preventing weight gain in African American breast cancer survivors using smart scales and activity trackers: A randomized controlled pilot study. J. Cancer Surviv..

[23] Kokts-Porietis R.L., Stone C.R., Friedenreich C.M., Froese A., McDonough M., McNeil J. (2019). Breast cancer survivors’ perspectives on a home-based physical activity intervention utilizing wearable technology. Support. Care Cancer.

[24] Nguyen N.H., Hadgraft N.T., Moore M.M., Rosenberg D.E., Lynch C., Reeves M.M., Lynch B.M. (2017). A qualitative evaluation of breast cancer survivors’ acceptance of and preferences for consumer wearable technology activity trackers. Support. Care Cancer.

[25] Gresham G., Schrack J., Gresham L.M., Shinde A.M., Hendifar A.E., Tuli R., Rimel B., Figlin R., Meinert C.L., Piantadosi S. (2018). Wearable activity monitors in oncology trials: Current use of an emerging technology. Contemp. Clin. Trials.

[26] Zeng N., Pope Z., Lee J.E., Gao Z. (2017). A systematic review of active video games on rehabilitative outcomes among older patients. J. Sport Health Sci..

[27] Moher D., Shamseer L., Clarke M., Ghersi D., Liberati A., Petticrew M., Shekelle P., Stewart L.A., PRISMA-P Group (2016). Preferred reporting items for systematic review and meta-analysis protocols (PRISMA-P) 2015 statement. Rev. Esp. Nutr. Hum. Diet..

[28] Pope Z., Zeng N., Gao Z. (2017). The effects of active video games on patients’ rehabilitative outcomes: A meta-analysis. Prev. Med..

[29] Pope Z., Lee J.E., Zeng N., Lee H.Y., Gao Z. (2019). Feasibility of smartphone application and social media intervention on breast cancer survivors’ health outcomes. Transl. Behav. Med..

[30] Dong X., Yi X., Gao D., Gao Z., Huang S., Chao M., Ding M. (2019). The effects of the combined exercise intervention based on internet and social media software (CEIBISMS) on quality of life, muscle strength and cardiorespiratory capacity in Chinese postoperative breast cancer patients: A randomized controlled trial. Health Qual. Life Outcomes.

[31] Vainshelboim B., Lima R.M., Myers J. (2019). Cardiorespiratory fitness and cancer in women: A prospective pilot study. J. Sport Health Sci..

[32] Joseph R.P., Keller C., Vega-López S., Adams M.A., English R., Hollingshead K., Hooker S.P., Todd M., Gaesser G.A., Ainsworth B.E. (2020). A culturally relevant smartphone-delivered physical activity intervention for African American women: Development and initial usability tests of smart walk. JMIR mHealth uHealth.

[33] Reeves M., Winkler E., McCarthy N., Lawler S., Eakin E., Healy G. (2012). Living well after breast cancer: Changes in objectively-measured physical activity in a weight loss trial. J. Sci. Med. Sport.

[34] Hartman S.J., Ms S.H., Myers E., Natarajan L., Sears D.D., Palmer B.W., Ba L.S.W., Parker B.A., Patterson R.E. (2018). Randomized controlled trial of increasing physical activity on objectively measured and self-reported cognitive functioning among breast cancer survivors: The memory & motion study. Cancer.

[35] Fawcett E., van Velthoven M.H., Meinert E. (2020). Long-term weight management using wearable technology in overweight and obese adults: Systematic review. JMIR mHealth uHealth.

[36] Dong X., Yi X., Ding M., Gao Z., McDonough D.J., Yi N., Qia W. (2020). A longitudinal study of a multicomponent exercise intervention with remote guidance among breast cancer patients. Intern. J. Environ. Res. Public Health.

[37] Neil-Sztramko S.E., Winters-Stone K.M., Bland K.A., Campbell K.L. (2019). Updated systematic review of exercise studies in breast cancer survivors: Attention to the principles of exercise training. Br. J. Sports Med..

[38] Su X., McDonough D.J., Chu H., Quan M., Gao Z. (2020). Application of network meta-analysis in the field of physical activity and health promotion. J. Sport Health Sci..

[39] Gao Z., Lee J. (2019). Emerging technology in promoting physical activity and health: Challenges and opportunities. J. Clin. Med..

[40] Latorre-Rojas E.J., Prat-Subirana J.A., Peirau-Terés X., Mas-Alòs S., Beltrán-Garrido J.V., Planas-Anzano A. (2019). Determination of functional fitness age in women aged 50 and older. J. Sport Health Sci..

[41] McDonough D.J., Su X., Gao Z. (2021). Health wearable devices for weight and BMI reduction in individuals with overweight/obesity and chronic comorbidities: Systematic review and network meta-analysis. Br. J. Sports Med..

